# CROSS-NATIONAL PREVALENCE OF RISK OF DEPRESSION AMONG OLDER ADULTS: ARE THERE GENDER AND AGE DIFFERENCES?

**DOI:** 10.1093/geroni/igae098.1159

**Published:** 2024-12-31

**Authors:** Antonia Diaz-Valdes Iriarte, Jose Medina

**Affiliations:** Universidad Mayor, Santiago, Region Metropolitana, Chile; Universidad Mayor, Santiago, Region Metropolitana, Chile

## Abstract

Depression among older adults is becoming one of the most pressing issues in public health, yet it remains one of the less recognized problems and is one of the leading causes of disability in this age group. Among older adults, depression often goes unrecognized. Although depression has been widely studied, there are still several unanswered questions regarding country/context differences, gender, age, and cohort differences, as most studies have been conducted in specific contexts. This study aims to examine cross-national gender gaps in the prevalence of depression risk from 2000 to 2020, controlling for age and cohort effects. A random-effects model with robust standard errors was conducted to examine depression cases and predict probabilities. Our analysis involved a sample of 248,559 individuals aged 50 and older from 35 countries, spanning the years 2000 to 2020. Throughout the examined period, the average predicted probability of depression was 24.01% for males and 35.70% for females. This translates to a consistent gender gap of 11.69 percentage points. Notably, while the gender gap in depression probability remained stable over time, both males and females exhibited an upward trend in depression probability during this period – about 3% and 5% respectively. This study contributes to the current literature and seeks to explore the prevalence of depression risk cross-nationally using harmonized measures of depression, which has not been done yet. Our study highlights the importance of screening and treating depression among older adults to reduce the consequences of depression, especially among women, who are disproportionately affected.

